# Snacking behaviours of adolescents and their association with skipping meals

**DOI:** 10.1186/1479-5868-4-36

**Published:** 2007-09-17

**Authors:** Gayle Savige, Abbie MacFarlane, Kylie Ball, Anthony Worsley, David Crawford

**Affiliations:** 1 Centre for Physical Activity and Nutrition Research, School of Exercise and Nutrition Sciences, Deakin University, Australia

## Abstract

**Background:**

Snacking is likely to play an important role in the development of overweight and obesity, yet little is known about the contexts of snacking in adolescents or how snacking may influence other dietary habits, like meal skipping. This study examines the contexts in which adolescents snack and whether these contexts are associated with demographic characteristics of adolescents and with meal skipping.

**Methods:**

A cross-sectional, self-reported online food habits survey was administered to 3,250 secondary students in years seven and nine. The students were drawn from 37 secondary schools in Victoria, Australia during 2004–2005. Frequencies of meal skipping, and snacking in eight contexts, were compared across gender, year level and region of residence. Logistic regressions were performed to examine associations between snacking contexts and meal skipping adjusting for gender and region.

**Results:**

The most common contexts for snacking among adolescents were after school (4.6 times per week), while watching TV (3.5 times per week) and while hanging out with friends (2.4 times per week). Adolescents were least likely to snack all day long (0.8 times per week) or in the middle of the night (0.4 times per week). Snacking contexts were variously associated with gender, year level and region. In contrast, meal skipping was associated with gender and region of residence but not year level. Adolescents who reported more frequent snacking on the run, on the way to or from school, all day long, or in the middle of the night were more likely to skip meals.

**Conclusion:**

These data suggest adolescents snack frequently, especially in their leisure time. In addition, adolescents who snack on the run, on the way to or from school, all day long or in the middle of the night are more likely to skip meals than are adolescents who don't snack at these times. Understanding the contexts in which adolescents snack, and their associations with skipping meals, may assist those involved in the promotion of healthy food habits among adolescents.

## Background

The rapid increase over the past three decades in the prevalence of childhood obesity in developed countries across the world [1] has led to increased concern about the diets of adolescents and children [2,3]. Although increased levels of sedentary behaviour are likely to be associated with this increase in obesity [4], changes in food consumption patterns are also likely to play an important role. Several dietary behaviours have been linked with adult and childhood obesity, including increased number of meals eaten outside the home [5], larger portion sizes of meals at restaurants and fast-food takeaways [6], and increased consumption of soft drinks [7,8]. The increasing prevalence of snacking has also recently been suggested as a potentially important influence on energy regulation in adolescents [9] and adults [10,11].

The prevalence of snacking (defined as *the consumption of foods and drinks between meals including milk drinks, regular soft drinks, sports drinks and energy drinks*) among adolescents and children varies widely across the world. For example, 87–88% of American adolescents (aged 12–18 years) consume at least one snack per day [12-14], with snacks contributing approximately 25% of their daily energy intake. In European countries, snacking is also highly prevalent, with Scottish adolescents (aged 15 years) consuming on average 2.8 snacks per day [15] and Portuguese youth (aged 5–15 years) consuming 1.5 snacks per day [16]. In Asian countries, snacking rates among youth (aged 2–19 years) are more variable. For example, in the Philippines, Russia and China, 86%, 71% and 10% of youth consume at least one snack on a daily basis, with snacks providing 18%,16% and 1% of their total daily energy, respectively [17].

Snacking is also commonly associated with undesirable health outcomes and dietary patterns. Since children and adolescents select snacks based on taste over nutrition, they more often choose salty, crunchy foods as snacks over healthier alternatives [12]. Consequently, snacking is commonly regarded as a contributing factor in the development of childhood overweight and obesity, although studies that have examined the association between snacking and body mass index have yielded mixed results [3,9,11,18]. Although evidence is limited, snacking may also be associated with less frequent consumption of meals, which may be detrimental to health since regular meal patterns are associated with greater dietary diversity [19], healthier food choices [20] and better nutrient intakes [21,22].

The literature exploring associations between snacking behaviour and demographic characteristics in children and adolescents is relatively scarce and contradictory. For example, the few studies that have examined the association between gender and snacking have yielded mixed results, with more frequent snacking reported among boys than girls [13,23] and *vice versa *[14], while some studies have failed to report gender differences at all [12,24]. In contrast, the few studies that have examined whether snacking varies with age, region of residence and socioeconomic status have yielded more consistent results. Snacking among children, adolescents and young adults occurs more often in younger than older subjects [12,13,16], more often in urban than rural residents [17], and more often in subjects from families with higher incomes [13,24,25] and education levels [13].

Surprisingly little is known about the context of snacking in adolescents, or how snacking may influence other dietary habits, such as meal skipping. While previous research shows that snacking among children and adolescents occurs most often in the afternoon [12,15,26] and at home [12,17], information about the specific contexts in which adolescents snack (e.g. while doing homework or working, while watching television) is lacking. Similarly, while meal skipping has been shown to be associated with a higher snacking frequency among both adolescents [22] and adults [11,27], to our knowledge no study has yet explored whether snacking is associated with a higher frequency of meal skipping.

The aim of the present study is to examine the contexts in which adolescents snack and to determine whether snacking contexts are associated with gender, year level, or region of residence. In addition, this study aims to examine whether snacking is associated with a higher frequency of meal skipping, and more specifically, which snacking contexts predict meal skipping.

## Methods

### Study procedure

For this study, we used cross-sectional data derived from the Youth Eating Patterns (YEP) survey, an online food habits survey conducted in secondary schools. The survey was administered during 2004 and 2005 and was approved by the Ethics committee of Deakin University and the Victorian Department of Education and Training and the Catholic Education Office. All co-educational state (government) and Catholic schools (including years 7 to 12), located in the Southern metropolitan region of Melbourne and the non-metropolitan region of Gippsland to the east of Melbourne, Australia, and with enrolments over 200, were invited to participate. Of the 70 schools (47 metropolitan and 23 non-metropolitan) that met these criteria, 37 schools (20 metropolitan and 17 non-metropolitan) agreed to participate in the study.

All students (*n *= 9,842) from year 7 (aged 12–13 years) and year 9 (aged 14–15 years) were invited to participate. Teachers distributed parental consent forms to parents via students asking permission for their child to participate in the study. Parental consent was obtained for 4,502 (46%) of all eligible students, but due to absence from school on the day of testing, teachers administered online surveys to 3,264 adolescents (73% of eligible students with parental consent and 33% of all eligible students that were invited to participate).

Teachers administered the online survey to students during a class when they had access to computers. Teachers instructed the students to type in the URL of the YEP survey, which was provided to teachers along with additional information covering answers to frequently asked questions and the procedure to re-commence the survey at a later time if students were unable to complete on the day. Further details of the sample and data collection procedures are described in a previous publication [28].

The present analyses are based on a subset of 3,250 students who completed sections of the survey on snacking behaviour.

### Measures

Prior to administration of the online survey, and as part of its development, the survey items were trialled among students from three classes in three separate schools (*n *= 50) and modified for clarity based on the students' feedback.

#### Snacking

Snacks were defined as *foods and drinks eaten between meals including milk drinks, regular soft drinks, sports drinks and energy drinks*. Snacking behaviour was assessed by asking adolescents how often over the past month they had snacked in the following eight contexts: 'after school', 'while watching television', 'while hanging out with friends', 'while doing homework or working', 'on the run' (on the way somewhere), 'on the way to or from school', 'all day long', and 'in the middle of the night'. Possible responses were 'not in the last month', 'once-twice a month', 'once-twice a week', 'most days', or 'every day'.

#### Meal skipping

Meal skipping was assessed by asking adolescents how often over the past month they had: 'skipped breakfast', 'skipped lunch', and 'skipped dinner'. Possible responses were 'not in the last month', 'once-twice a month', 'once-twice a week', 'most days', or 'every day'.

### Statistical analyses

The data were analysed using SPSS version 12.0. Descriptive statistics were used to describe the snacking and meal skipping habits of adolescents. Differences in snacking and meal skipping frequencies in relation to year level, sex and region of residence were analysed using the chi-square statistic and the categories responsible for significant χ^2 ^values were identified by examining the standardized residuals [29].

The mean number of times adolescents snacked per week was calculated by converting response categories to a weekly equivalent (e.g. once-twice a month = 0.05 times/week; once-twice a week = 1.5; most days = 5 times/week).

Logistic regression was used to examine whether eight different snacking contexts (predictor variables) were associated with skipping breakfast, lunch or dinner (outcome variables). Since snacking contexts were not mutually exclusive, adolescents could include the same snacking behaviour in more than one context (e.g. 'after school', 'while hanging out with friends' and 'while watching television'). Consequently, the logistic regression analyses were performed separately for each snacking context. Since our chi-square analyses showed that sex and region of residence were associated with meal skipping, all models adjusted for these factors. For each snacking context, adolescents were classified into one of four groups: 'non-snackers' (not snacked in the last month), 'low snackers' (snacked once-twice a month), 'medium snackers' (snacked once-twice a week) and 'high snackers' (snacked most days or every day). Adolescents who skipped breakfast, lunch or dinner most days or more were classified as 'meal skippers'. A *p *value of ≤ 0.01 was considered significant.

## Results

### Characteristics of the sample

The sociodemographic characteristics of the sample are presented in Table 1. Among the 3,250 adolescent participants, who ranged in age from 12 to 15 years, more were female than male, more were in year 7 than 9, and more resided in the metropolitan than the non-metropolitan region of Victoria.

**Table 1:** Demographic characteristics

	%
Sex	(*n *= 3250)
Male	47
Female	53
	
Year level	(*n *= 3250)
Year 7 (12–13 years)	62
Year 9 (14–15 years)	38
	
Region of Victoria	(*n *= 3250)
Metropolitan	67
Non-metropolitan	33

### Snacking

The frequency of snacking among adolescents across eight snacking contexts is shown in Table 2. Snacking after school was the most common context for snacking (4.6 times per week) with 75% of adolescents indicating they snacked during this time on most days or every day. Adolescents also snacked frequently while watching TV and while hanging out with friends (3.5 and 2.4 times per week on average, respectively). Snacking while doing homework or working, on the run, or on the way to or from school was less common, occurring 1.8, 1.3 and 1.0 times per week, respectively. Adolescents were least likely to snack all day long (0.8 times per week) or in the middle of the night (0.4 times per week) with less than 21% of adolescents reporting that they often (once-twice a week or more) snacked in these contexts.

**Table 2:** Frequency of snacking among adolescents in eight snacking contexts according to year level, gender and region

**Snacking context**	**M (± SD) snacking/week^a^**	**Total %**	**Year level %**			**Gender %**			**Region %**		
***n *= 3250**			**7**	**9**		**Male**	**Female**		**Metro**	**Non-Metro**	

**After school**	4.6 (± 2.4)										**
*not in last month*		4	4	3		4	3		4	3	
*once/twice a month*		7	8	7		8	7		8	5	
*once/twice a week*		14	14	14		14	14		15	13	
*most days*		41	39	43		39	43		40	42	
*every day*		34	35	33		35	33		33	37	
**While watching TV**	3.5 (± 2.5)										**
*not in last month*		7	8	6		9	6		7	9	
*once/twice a month*		12	12	11		12	12		12	12	
*once/twice a week*		26	25	28		24	27		24	29	
*most days*		39	39	39		38	39		40	35	
*every day*		16	16	16		17	15		17	15	
**While hanging out with friends**	2.4 (± 2.5)				**			**			
*not in last month*		14	16	11		17	12		15	13	
*once/twice a month*		25	24	27		24	27		25	25	
*once/twice a week*		24	22	27		24	24		23	26	
*most days*		27	27	26		25	27		27	25	
*every day*		10	11	9		10	10		10	11	
**While doing homework or working**	1.8 (± 2.3)							**			*
*not in last month*		29	30	28		36	24		30	28	
*once/twice a month*		20	19	21		19	21		19	21	
*once/twice a week*		23	23	24		20	26		22	26	
*most days*		22	22	21		19	24		23	19	
*every day*		6	6	6		6	5		6	6	
**On the run**	1.3 (± 2.0)							**			
*not in last month*		28	30	25		31	25		29	26	
*once/twice a month*		30	30	31		28	32		30	31	
*once/twice a week*		24	23	26		22	26		23	26	
*most days*		14	14	15		14	14		14	14	
*every day*		4	4	4		5	3		4	4	
**On the way to or from school**	1.0 (± 2.0)							*			
*not in last month*		57	56	59		56	57		57	56	
*once/twice a month*		17	17	17		17	17		17	17	
*once/twice a week*		12	13	11		11	13		12	13	
*most days*		10	10	8		10	10		10	9	
*every day*		4	4	5		6	3		4	5	
**All day long**	0.8 (± 1.8)				**						
*not in last month*		63	67	56		63	63		62	65	
*once/twice a month*		16	14	20		15	16		16	16	
*once/twice a week*		10	9	12		10	11		11	10	
*most days*		8	7	8		8	7		8	6	
*every day*		3	3	4		4	3		3	3	
**In middle of night**	0.4 (± 1.4)							**			**
*not in last month*		80	81	78		75	84		77	85	
*once/twice a month*		9	9	10		10	9		11	6	
*once/twice a week*		5	4	5		6	3		5	4	
*most days*		4	4	4		5	3		4	3	
*every day*		2	2	3		4	1		3	2	

Snacks are defined as foods and/or drinks that are consumed outside the main meals of breakfast, lunch and dinner.

^a ^Mean (± SD) times per week adolescents (*n *= 3,250) snacked in each snacking context.

* p ≤ 0.01, ** p ≤ 0.001. Pearson's chi square test of significance.

The contexts in which adolescents snacked were associated with year level, sex and region of residence (Table 2). In terms of year level, adolescents in Year 9 were more likely than those in Year 7 to report that they often (several times a week to several times a month) snacked while hanging out with friends or all day long.

Several snacking contexts were also associated with gender (Table 2). Females were more likely than males to report that they often (most days to several times a month) snacked on the run, while hanging out with friends and while doing homework or working. In contrast, males were more likely than females to report daily snacking on the way to or from school and in the middle of the night.

Snacking contexts were also associated with region of residence (Table 2). Adolescents from metropolitan regions were more likely than those from non-metropolitan regions to report that they often snacked in the middle of the night (once-twice a month or more), while doing homework or working (most days) and while watching TV (most days). In contrast, non-metropolitan adolescents were more likely to report that they snacked every day after school.

### Meal skipping

On a daily basis, more adolescents skipped breakfast (20%) than skipped lunch (12%) or dinner (2%) (Table 3). Meal skipping was associated with gender and region of residence, but not year level (Table 3). Females were more likely than males to report skipping breakfast and lunch on most days or every day in the past month. Similarly, adolescents from metropolitan regions were more likely to report skipping breakfast on most days or every day in the past month, compared with non-metropolitan adolescents.

**Table 3:** Frequency of meal skipping among adolescents according to year level, sex and region

**Meal**	**Total**	**Year level**			**Gender**			**Region**		
***n *= 3,250**		**7**	**9**		**Male**	**Female**		**Metro**	**Non-Metro**	

**Breakfast**							**			**
*Skips*	20	19	21		16	23		21	17	
*Eats*	80	81	79		84	77		79	83	
										
**Lunch**							*			
*Skips*	12	11	12		10	13		12	10	
*Eats*	88	89	88		90	87		88	90	
										
**Dinner**										
*Skips*	2	2	2		2	2		3	1	
*Eats*	98	98	98		98	98		97	99	

Values are presented as %

Skips = skips a meal most days or every day

Eats = skips a meal once-twice a week or less

* p ≤ 0.01, ** p ≤ 0.001. Pearson's chi square test of significance.

### Snacking and meal skipping

Logistic regression analyses were performed to predict the odds of adolescents skipping meals when snacking in different contexts (Table 4). Adolescents who frequently snacked on the run, on the way to or from school, all day long or in the middle of the night were at greater risk of skipping meals. For example, compared to those adolescents who did not snack, the likelihood of skipping breakfast was higher among adolescents who frequently (most days or every day) snacked while doing homework or working (OR = 1.3), on the run (OR = 2.5), on the way to or from school (OR = 2.2), all day long (OR = 2.0) or in the middle of the night (OR = 3.9). Similarly, compared to adolescents who did not snack, the likelihood of skipping lunch was higher among adolescents who frequently snacked on the run (OR = 1.9), on the way to or from school (OR = 2.1), all day long (OR = 1.9) or in the middle of the night (OR = 3.7). Finally, the likelihood of skipping dinner was higher among adolescents who frequently snacked on the way to or from school (OR = 3.3), all day long (OR = 3.3) or in the middle of the night (OR = 5.4), compared to adolescents who did not snack in these contexts.

**Table 4:** Adjusted odds ratios (OR) and 95% confidence intervals (CI) for snacking in eight snacking contexts and the likelihood of skipping meals among adolescents

	**Breakfast**			**Lunch**			**Dinner**		
	
**Snacking context**	**OR**	**95% CI**		**OR**	**95% CI**		**OR**	**95% CI**	
**After school**									
*Non-snacker*	1.00			1.00			1.00		
*Low snacker*	0.69	0.41–1.16		0.87	0.44–1.70		1.01	0.37–2.77	
*Medium snacker*	0.78	0.49–1.24		0.77	0.41–1.43		0.48	0.18–1.34	
*High snacker*	0.66	0.43–1.01		0.94	0.54–1.65		0.36	0.15–0.86	
**While watching TV**									
*Non-snacker*	1.00			1.00			1.00		
*Low snacker*	0.68	0.44–1.05		0.80	0.47–1.35		0.52	0.20–1.36	
*Medium snacker*	0.85	0.59–1.24		0.87	0.55–1.38		0.31	0.12–0.77	*
*High snacker*	1.25	0.88–1.77		1.14	0.74–1.74		0.64	0.31–1.32	
**While hanging out with friends**									
*Non-snacker*	1.00			1.00			1.00		
*Low snacker*	0.96	0.70–1.30		0.36	0.59–1.21		0.49	0.23–1.04	
*Medium snacker*	1.24	0.92–1.68		0.94	0.69–1.41		0.48	0.22–1.04	
*High snacker*	1.38	1.04–1.83		0.69	0.77–1.49		0.84	0.45–1.57	
**While doing homework or working**									
*Non-snacker*	1.00			1.00			1.00	0.50–2.25	
*Low snacker*	0.72	0.55–0.95		0.69	0.49–0.98		1.06	0.44–1.97	
*Medium snacker*	1.01	0.79–1.29		0.96	0.71–1.30		0.93	1.08–3.61	
*High snacker*	1.33	1.06–1.66	*	1.39	1.06–1.82		1.97	0.58–1.48	
**On the run**									
*Non-snacker*	1.00			1.00			1.00		
*Low snacker*	1.02	0.80–1.31		1.04	0.77–1.40		0.42	0.20–0.86	
*Medium snacker*	1.33	1.03–1.71		1.20	0.88–1.63		0.69	0.35–1.34	
*High snacker*	2.48	1.92–3.19	**	1.88	1.38–2.55	**	1.52	0.85–2.72	
**On way to or from school**									
*Non-snacker*	1.00			1.00			1.00		
*Low snacker*	1.49	1.18–1.89	**	0.95	0.69–1.30		1.05	0.49–2.23	
*Medium snacker*	1.49	1.14–1.94	*	1.17	0.83–1.64		1.79	0.89–3.62	
*High snacker*	2.24	1.77–2.85	**	2.11	1.60–2.78	**	3.29	1.88–5.75	**
**All day long**									
*Non-snacker*	1.00			1.00			1.00		
*Low snacker*	1.26	0.99–1.61		1.15	0.85–1.56		1.37	0.69–2.74	
*Medium snacker*	1.63	1.24–2.14	**	1.40	1.00–1.97		2.14	1.07–4.28	
*High snacker*	1.98	1.53–2.57	**	1.85	1.35–2.52	**	3.26	1.81–5.89	**
**In middle of night**									
*Non-snacker*	1.00			1.00			1.00		
*Low snacker*	1.50	1.13–2.0	*	1.50	1.06–2.12		2.29	1.16–4.54	
*Medium snacker*	1.89	1.29–2.77	**	1.62	1.01–2.61		2.64	1.09–6.36	
*High snacker*	3.92	2.89–5.32	**	3.66	2.61–5.14	**	5.37	2.91–9.89	**

High snackers included adolescents who snacked 'most days' or 'everyday'. Adjusted for sex and region of residence.

* p ≤ 0.01, ** p ≤ 0.001.

## Discussion

This study explored the contexts in which adolescents snack and whether these contexts were associated with demographic characteristics of adolescents and with skipping meals. Adolescents most frequently snacked after school, while watching TV, and while hanging out with friends. They snacked less frequently while doing homework or working, on the run and on the way to or from school, but were least likely to snack all day long and in the middle of the night. While these snacking contexts were variously associated with gender, year level and region of residence, meal skipping was only associated with gender and year level. Adolescents who reported more frequent snacking on the run, on the way to or from school, all day long, or in the middle of the night were more likely to skip meals.

The finding that adolescents snacked most frequently after school is consistent with previous research showing that children and adolescents snack most often in the afternoon [12,15,26]. The reasons for this are likely to be physiological, as well as related to school policy in Australia. Given that adolescents have high energy demands due to rapid growth and development [30], and given the typical Australian lunch is usually a light, uncooked meal (e.g. sandwich) brought from home, students are likely to be hungry after school. In addition, snacking is not usually permitted in class time and consequently the first opportunity students have to snack (following the lunch break) is after school. Adolescents also snacked frequently while watching TV, which is not surprising given that television viewing has previously been shown to be positively associated with snacking [31-33].

Snacking frequencies in different contexts varied according to the demographic characteristics of adolescents. Year level was positively associated with the frequency of snacking in two contexts; adolescents in year 9 were more likely than their younger peers in year 7 to report that they often snacked while hanging out with friends and all day long. These findings contrast with previous studies that have found a decline in snacking as children become older [12,13,16], however these studies covered a wider range of ages and compared overall snacking frequencies.

Several snacking contexts were associated with gender but the direction of these associations was mixed. Boys were more likely than girls to report that every day they snacked on the way to or from school and in the middle of the night, whereas girls were more likely to report that they often snacked on the run, while hanging out with friends and while doing homework or working. The mixed direction of these associations is consistent with previous studies on gender differences in snacking, which also yielded mixed results [12-14,23,24]. However, the higher reported frequency of snacking among girls while hanging out with friends and while doing homework or working may reflect the greater time girls spend with their friends [34] and doing homework [33,35]. That boys were more likely than girls to snack in the middle of the night is consistent with a recent study of the eating patterns of Swedish adolescents, which also found boys eat more throughout the night than girls [36]. This study also reported that more boys than girls eat between 0600 and 1000 hours, which is consistent with our finding that boys were more likely than girls to snack on the way to school.

Several snacking contexts were associated with region of residence. Adolescents from metropolitan regions were more likely than non-metropolitan adolescents to report that they often snacked in the middle of the night, while doing homework or working and while watching TV. A higher frequency of snacking among children and adolescents (aged 2–18 years) from urban areas has also been reported in several other countries, including China, Russia, the US and the Philippines [17]. In our study, however, we also found that adolescents from non-metropolitan areas were more likely than their peers from metropolitan areas to report that they snacked every day after school. Adolescents from non-metropolitan areas may have more opportunity to snack after school since a larger proportion are transported directly home from school via a school bus. In contrast, metropolitan students have greater flexibility regarding their transport options home. This enables them greater opportunities to pursue activities outside the home, which may restrict their access to snacks, but also their snacking behaviour as a result of the activity *per se*.

Meal skipping was associated with gender and region of residence. Females were more likely than males to skip breakfast and lunch. Similarly, adolescents from metropolitan areas were more likely than their peers from non-metropolitan areas to skip breakfast. Previous studies have also reported a higher frequency of breakfast skipping among female adolescents [37,38]. In addition, a recent study of meal skipping patterns among fourth grade children from distinct geographical locations in Maryland, USA, found that urban students were more likely to skip breakfast compared with suburban and rural students [39].

This study also assessed the association of snacking in different contexts with the skipping of meals. Adolescents who frequently snacked on the run, on the way to or from school, all day long, or in the middle of the night were more likely to skip meals. In one Australian study, the two major reasons adolescents most commonly reported for skipping breakfast included a lack of time in the morning (52%) and not being hungry (22%) [38]. Snacking on the run and on the way to or from school are behaviours that could be associated with a lack of time and support our finding that snacking in these contexts increased the likelihood of adolescents skipping breakfast, compared with adolescents who did not snack in these contexts. By snacking in the middle of the night adolescents reduce the length of the overnight fasting period, which may diminish their sense of hunger, and thereby increase their likelihood of skipping breakfast. Snacking all day long is also likely to affect hunger at subsequent meals.

Interestingly, the contexts in which adolescents most commonly snacked (i.e. after school, while watching TV, while hanging out with friends, while working or doing homework) were not associated with skipping meals, while those contexts in which adolescents least commonly snacked (i.e. on the run, on the way to or from school, all day long and in the middle of the night) were associated with skipping meals. Snacking all day long and in the middle of the night may be regarded as the most health-compromising of the contexts we considered. Our finding that these contexts were those associated with meal skipping (a mildly disordered eating behaviour) is consistent with several previous cross-sectional studies, which found strong associations between breakfast skipping and various health compromising behaviours (e.g. smoking, alcohol use and sedentary lifestyle) among adults and adolescents [37] and between infrequent meal patterns and disordered eating (e.g. unhealthy weight control behaviours, binge-eating and chronic dieting) among adolescents [40]. These associations, however, should be treated cautiously since only 6% and 11% of adolescents were high snackers in the middle of the night and all day long, respectively.

Strengths of this study include the large and diverse nature of our study population. To our knowledge, this is the largest survey on snacking behaviour of adolescents in Australia. A limitation of our study was its cross-sectional nature, which limits our ability to discuss directionality and causality. We cannot assume that snacking in certain contexts (e.g. in the middle of the night, on the way to or from school) precedes meal skipping. It is equally probable that adolescents who skip meals are more likely to snack in these various contexts, compared with adolescents who do not skip meals. Also, since we did not ask students to indicate what foods or drinks they consumed as snacks, we are unable to determine if the nutritional quality of the snacks varied according to snacking context.

The findings point to a need for further research studying associations between snacking contexts and meal skipping. Future research should aim to employ longitudinal or experimental designs to clarify directionality and provide additional insight into possible causal mechanisms. Future research should also examine whether adolescents consume different types of snacks in different snacking contexts. For example, are adolescents more likely to snack on chips and chocolate (energy dense foods) while watching TV, fruit while doing homework or working, and milk in the middle of the night? Furthermore, does the healthfulness (or energy content) of the snack predict the likelihood of skipping meals. For example, does the consumption of 'unhealthy' or energy dense snacks increase the likelihood of skipping meals? Additionally, is body mass index associated with snacking in different contexts? For example, are overweight or obese adolescents more likely to snack in the middle of the night or all day long, compared with adolescents in the healthy weight range?

## Conclusion

Energy and nutrient requirements are greatly increased in adolescence to accommodate the rapid growth and development that occurs during this period [30]. The rising incidence of obesity among adolescents, however, indicates that many youth are consuming more than adequate intakes of energy. Since snacks eaten between meals provide up to a quarter of the daily energy intake in some adolescent populations, limiting snacks may be an effective way for adolescents to reduce their total energy intake. However, given that snacking is such a common dietary behaviour among adolescents, promotion of nutritious snacks (e.g. nuts, fresh fruit and vegetables, bread/toast, milk and pure fruit juice) is crucial. Furthermore, promoting nutritious snacks to those adolescents that skip breakfast may be more useful than encouraging the consumption of breakfast (either at home or school), especially if a lack of time (perceived or otherwise) is the cause of this behaviour. Understanding the contexts in which adolescents snack, and their relationships with meal skipping, may help parents and health promotion officers develop strategies to promote healthy food habits among adolescents.

## Competing interests

The author(s) declare that they have no competing interests.

## Authors' contributions

GS assisted in the development of the survey instrument, analysed the data and conceived and drafted the original manuscript with assistance from TW. AM performed additional analyses and substantially revised the original manuscript. KB, DC and TW designed the overall study project, and provided critical feedback on drafts. All authors read and approved the final manuscript.
